# Use of Eltrombopag for the First Trimester Pregnancy Complicated with Refractory Idiopathic Thrombocytopenic Purpura: A Case Report and Literature Review

**DOI:** 10.7759/cureus.22505

**Published:** 2022-02-22

**Authors:** Satoshi Shibata, Takuya Misugi, Takahiko Nakane, Masayuki Hino, Daisuke Tachibana

**Affiliations:** 1 Obstetrics and Gynecology, Osaka City University Graduate School of Medicine, Osaka, JPN; 2 Hematology, Osaka City University Graduate School of Medicine, Osaka, JPN

**Keywords:** refractory thrombocytopenia, thrombopoietin receptor agonist, eltrombopag, pregnancy, idiopathic thrombocytopenic purpura

## Abstract

Severe idiopathic thrombocytopenic purpura (ITP) is thought to occur in approximately one in 10,000 pregnancies and refractoriness for conventional treatment is life-threatening. We herein describe a pregnant case of refractory ITP successfully treated with eltrombopag, orally medicated thrombopoietin receptor agonist (TPO-RA). The patient was diagnosed with ITP at the age of 22 and corticosteroid, immune globulin treatments, or splenectomy was invalid. Eltrombopag was administered at 25 mg/day and the platelet count was maintained at 50-80 × 10^9^/L. At the age of 32, she discontinued the medication out of fear of the possible adverse events of TPO-RA. She became pregnant and visited our hospital at the gestational age of 9th week with the result of a platelet count at 18 × 10^9^/L. Two weeks later, her platelet counts further decreased to 9 × 10^9^/L, and eltrombopag administration was resumed with 12.5 mg/day after informed written consent. The platelet counts subsequently increased and were maintained between 30 and 90 × 10^9^/L. At 34th gestational week, the dose of eltrombopag was increased to 25 mg/day in preparation for delivery and the count increased to 123 × 10^9^/L by the 38th gestational week. A healthy female infant weighing 2370 g was vaginally delivered by induced labor. Maternal blood loss was 0.4 L at delivery, and the newborn’s platelet count was 173 × 10^9^/L on the second day after birth and 174 × 10^9^/L on the fifth day. The infant is neurologically and developmentally intact at 6 months of age. Regarding TPO-RA treatment for refractory ITP during pregnancy, only six pregnant cases including the present one treated with eltrombopag have been reported so far. This case is rare with reference to the use of eltrombopag for the first trimester and we increased the dose of eltrombopag at the gestational age of 34 in preparation for delivery and vaginal delivery was successful without platelet transfusion. Despite the very limited information on eltrombopag, its use might be a possible optional treatment for refractory ITP during pregnancy.

## Introduction

Idiopathic thrombocytopenic purpura (ITP) is an autoimmune disease characterized by both increased platelet destruction and reduced platelet production [1]. Severe ITP (platelets count less than 50 × 10^9^/L) is one of the most challenging diseases during pregnancy and is thought to occur in approximately one in 10,000 pregnancies [2]. Furthermore, refractory ITP cases for conventional first-line treatment, i.e., corticosteroids, intravenous immunoglobulin, and splenectomy are relatively rare in the obstetrical practice, however, this condition is life-threatening for the pregnant woman [3]. Recent works of literature on thrombopoietin receptor agonist (TPO-RA) report its efficacy and safety for refractory ITP in a relatively large population and randomized studies [4], however, its use during pregnancy and the effect on the neonatal outcome is limited. We herein describe a pregnant case of refractory ITP successfully treated with orally medicated TPO-RA (eltrombopag) and we also review the literature of its use during pregnancy.

## Case presentation

**Figure 1:**
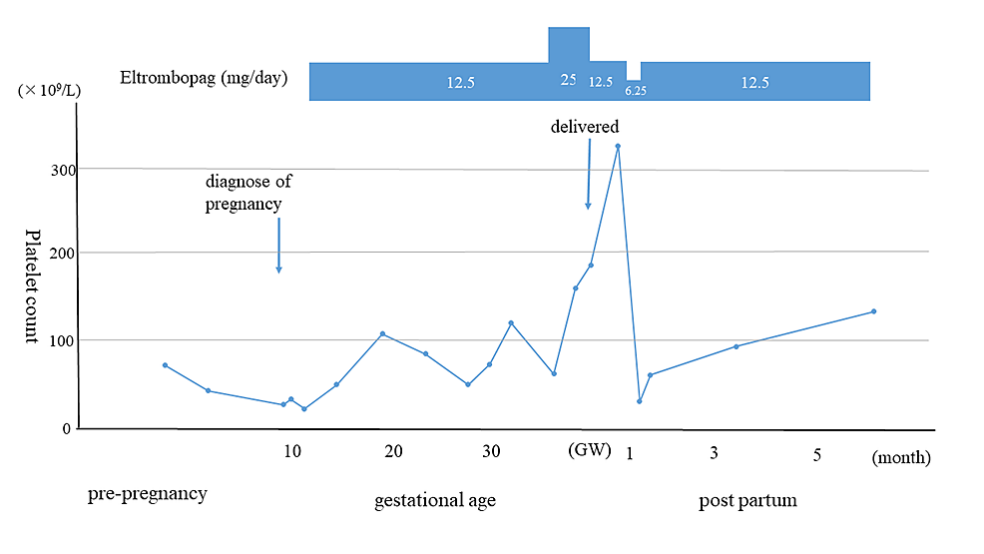
The transition of platelet count before, during, and after pregnancy. In this pregnancy, she first administered eltrombopag at 11th gestational week. Platelet counts were kept above 30 × 10^9^/L during pregnancy. In preparation for the delivery, the dose of eltrombopag was increased to 25 mg/day at the 36th gestational week.

The patient was diagnosed with ITP after thrombocytopenia was discovered by an occasional medical examination at the age of 22. The test of *Helicobacter pylori* was negative and corticosteroid and immune globulin treatments did not increase platelet counts. At the age of 24, splenectomy was undergone with platelet transfusion, however, platelet count gradually decreased after a few years. Eltrombopag was administered 25 mg/day and the platelet was maintained at 50-80 × 10^9^/L. At the age of 32, she married and discontinued the medication by her own request for the fear of the teratology of TPO-RA. She became pregnant after 9 months and visited our department at the gestational age of 9th week with the result of platelet count 18 × 10^9^/L. Two weeks later platelet count further decreased to 9 × 10^9^/L and eltrombopag administration was resumed with 12.5 mg/day after informed written consent. The platelet count increased and was maintained between 30 and 90 × 10^9^/L (Figure 1). At 34th gestational week, the dose of eltrombopag was increased to 25 mg/day in preparation for delivery and platelet increased to 123 × 10^9^/L at 38th gestational week. A healthy female infant weighing 2370 g was vaginally delivered by induced labor. Maternal blood loss was 0.4 L at delivery and the newborn’s platelet count was 173 × 10^9^/L on the second day after birth and 174 × 10^9^/L on the fifth day. Breastfeeding was not recommended as the effects of eltrombopag on the neonate are unknown. The maternal platelet count after delivery was 119 × 10^9^/L and eltrombopag was reduced to 12.5 mg/day. The patient was discharged from our hospital with her baby on the fifth postpartum day. The infant is neurologically and developmentally intact so far at the age of 6 months.

## Discussion

TPO-RA stimulates TPO-receptor of bone marrow to increase the production of platelets and two TPO receptor agonists have been approved: romiplostim and eltrombopag. Romiplostim, a peptide with molecular weight of 59,085 Dalton, is administered subcutaneously binding to the extracellular TPO receptor, while eltrombopag, a non-peptide with molecular weight of 546 Dalton, is administered orally binding to a transmembrane site of the TPO receptor, both of them promote the differentiation and proliferation of megakaryocyte and increase the production of platelets [5,6]. Romiplostim or eltrombopag have been investigated in several randomized controlled trials involving adult and pediatric ITP patients [7-9], and meta-analysis reports show that TPO receptor agonists with ITP patients were an effective second-line treatment option for primary ITP patients, in whom at least one previous therapy has failed [10]. Among patients treated with romiplostim, an average of 75% showed a significant response in platelet counts, whereas 69% of patients responded with eltrombopag [11]. An initial response to TPO-RA commonly appears within 1 to 2 weeks. If one TPO-RA agent is ineffective, switching to the other leads to a platelet response in up to 50% of patients [11,12].

In this case, her platelet count decreased to 9 × 10^9^/L and we administered eltrombopag at the gestational age of 11 weeks to avoid frequent platelet transfusion. Regarding TPO-RA treatment during pregnancy, however, there is no international consensus on the optimal treatment [13] and the use of eltrombopag is quite limited. As far as we researched, only five pregnant cases treated with eltrombopag have been reported. The summary of the cases including the present case is shown in Table 1. Two cases were complicated other than ITP. One thrombocytopenia case accompanied by systemic lupus erythematosus (SLE) was firstly reported by Alkaabi et al. [14]. The case did not respond to eltrombopag 50 mg/day, later increased to 75 mg/day, and further switched to romiplostim, which showed a significant increase of platelets [14]. Favier et al. medicated eltrombopag from 36th gestational week to a case complicated with autosomal dominant thrombocytopenia, i.e., *MYH9*-related disease, and successful platelet increase was observed [15]. Four cases of ITP, including the present case, were treated with eltrombopag (cases 3-6) [16-18]. Case 3 showed slight platelet increase necessitated with platelet transfusion for vaginal delivery and case 4 achieved platelet increase over 60 × 10^9^/L, however, platelet count fell to 19 × 10^9^/L at 36th week of gestation, where preeclampsia could not be ruled out from the cause of underlying pathology [16], and the patient subsequently needed platelet transfusion for delivery. Sufficient platelet increases with eltrombopag were observed in cases 4 and 6 (our case) [17]. In particular, we increased the dose of eltrombopag at the gestational age of 34 and vaginal delivery was succeeded without platelet transfusion. The impact of TPO-RA on the fetus is unknown due to no basic or clinical study being reported about the placental passage of TPO-RA. No neonate showed a congenital anomaly, although the two cases were medicated before pregnancy (case 5) or from the first trimester (case 6) [18]. Birth weights were relatively light-for-date, however, it is very difficult to mention whether it is resulted from the medication or maternal complication. No case showed maternal liver enzyme elevation or abnormal platelet count including thrombocytosis of the neonates.

**Table 1:** Cases of thrombocytopenia treated with eltrombopag during pregnancy. P: primiparous; M: multiparous; SLE: systemic lupus erythematosus; PE: preeclampsia;  CS: cesarean section; GW: gestational age (weeks); PSL: prednisolone; ITP: idiopathic thrombocytopenic purpura; BW: birth weight

	Author	Age P/M	Maternal disease	Drug (dose)	Commencement	Platelet count (×10^9^/L)		Delivery			Neonate		
						Commencement	At delivery	GW	Mode of delivery	Transfusion	BW (g)	Platelet count (×10^9^/L)	Anomaly
1	Alkaabi et al. [14]	34 M	SLE	Eltrombopag (50-75 mg/day)	30 weeks	2-5	52	34 weeks	Induced vaginal	None	No data	Normal	None
				Romiplostim (3-6 μg/kg)	34 weeks								
2	Favier et al. [15]	41 M	*MYH0*-related disease	Eltrombopag (50 mg/day)	36 weeks	30	162	39 weeks	CS (breech presentation)	None	3145	62	None
3	Purushothaman et al. [16]	27 M	ITP	Eltrombopag (25-50 mg/day)	29 weeks	10	30	36 weeks	Vaginal	Platelet transfusion	1860	55	None
4	Ferreira et al. [17]	33 P	ITP	Eltrombopag (25-50 mg/day + tapered PSL)	27 weeks	40	258	37 weeks	Induced vaginal (PE)	none	2400	282	None
5	Suzuki et al. [18]	25 P	ITP	Eltrombopag (12.5 mg/day + PSL)	Before pregnancy	-	19	37 weeks	CS (PE)	Platelet transfusion	1670	416	None
6	Present case	32 P	ITP	Eltrombopag (12.5-25 mg/day)	11 weeks	9	123	38 weeks	Induced vaginal	None	2370	173	None

A threshold for initiation of treatment in the non-pregnant condition is recommended platelet count below 20 × 10^9^/L, even in the absence of bleeding, since severe bleeding events such as intracerebral hemorrhage might occur at not low frequency [19]. Raising the platelet count is recommended in cases with comorbidities, older age, and exposure to antiplatelet or anticoagulant drugs [19]. Owing to the lack of evidence-based guidelines, empirical suggestion recommends the maintenance of the platelet count above 20 × 10^9^/L to 30 × 10^9^/L during pregnancy [20]. In our case, her platelet counts were kept above 30 × 10^9^/L during pregnancy by administering eltrombopag and successfully delivered without platelet transfusion.

## Conclusions

Despite the very limited information of eltrombopag during pregnancy, we conclude as follows: first, four thrombocytopenia cases out of six cases refractory to previous medication responded to an increase in the platelet count; second, no congenital anomaly or abnormal hematological finding of neonatal blood was observed, including the two cases in which medication was already started before pregnancy or in the first trimester, despite the slight possible adverse effect for fetal growth. Further studies should be needed to elucidate the efficacy and safety of eltrombopag during pregnancy to improve maternal and neonatal outcomes.
